# Increased mean carotid intima media thickness in type 2 diabetes mellitus patients with non-blood pressure component metabolic syndrome: A preliminary report

**DOI:** 10.4103/0973-3930.50710

**Published:** 2009

**Authors:** Marwan S. M. Al-Nimer, Ismail I. Hussein

**Affiliations:** Department of Pharmacology, College of Medicine, Al-Mustansiriyia University, P.O. Box 14132, Baghdad, Iraq; 1 Department of Physiology, College of Medicine, Al-Mustansiriyia University, P.O. Box 14132, Baghdad, Iraq

**Keywords:** Intima media thickness, metabolic syndrome, type 2 diabetes mellitus

## Abstract

**AIMS:**

Patients with type-2 diabetes mellitus have greater carotid intima media thickness and they are at risk for generalized atherosclerosis. This study aimed to compare the thickness of carotid artery intima media in type-2 diabetes mellitus patients with and without nonblood pressure component metabolic syndrome.

**SETTINGS AND DESIGN:**

This was a comparative observational study conducted in the Departments of Pharmacology and Physiology in the College of Medicine, Al-Mustansiriyia University in cooperation with Baghdad Teaching Hospital.

**MATERIALS AND METHODS:**

Forty-six diabetic patients of both sexes with systolic blood pressure < 130 mm Hg and diastolic blood pressure < 85 mm Hg were subjected to high resolution B-mode ultrasonography of the common and internal carotid arteries. Patients were grouped into those without metabolic syndrome (Group I) and with nonblood pressure component metabolic syndrome (Group II).

**STATISTICAL ANALYSIS:**

The two-tailed unpaired Student's t-test was used in this study.

**RESULTS:**

Significantly high mean thickness was observed in the common carotid intima media (0.824 ± 0.155 mm) but not in the internal carotid arteries in group II patients compared to group I patients (0.708 ± 0.113 mm). Group II also had a significant number of patients with increased lesion intima media thickness (≥ 1.1 mm).

**Conclusion:**

The greater carotid intima media thickness observed in type 2 diabetes mellitus patients is related to the metabolic syndrome even in the absence of the blood pressure component.

## Introduction

Measurement of carotid intima media thickness (IMT) using high-resolution B-mode ultrasonography is a noninvasive, well validated method to assess early cardiovascular disease.[1,2] In type-2 diabetes mellitus (T2DM), carotid IMT is significantly higher than in corresponding healthy, age- and sex-matched nondiabetic subjects.[3,4]

Hypertension, duration of diabetes, hyperglycemia, and dyslipidemia are associated with IMT and have been identified as significant risk factors for stroke.[5,6] Increased IMT is associated with increased risk of stroke[7] as well as the risk of incident stroke.[8] Common carotid artery IMT in patients with diabetes and acute stroke is significantly greater than in stroke-free patients with diabetes.[9] Earlier studies showed that insulin resistance, an important feature of metabolic syndrome in T2DM, did not correlate with IMT.[3,10] Hassinen *et al.* later found that after adjustment of other risk factors, the increase in carotid IMT was greater in elderly women who developed metabolic syndrome than in those who did not.[11] Bertoni *et al.* found that the nonglucose component of metabolic syndrome did not correlate to increased IMT.[12] Moreover, reducing the systolic blood pressure to ≤115 mm Hg in type-2 diabetes mellitus patients resulted in the regression of carotid IMT.[13]

This study aimed to demonstrate the association of increased carotid artery IMT with nonblood pressure component metabolic syndrome in T2DM patients.

## Materials and Methods

Subjects in this investigation were recruited from the vascular Doppler unit in Baghdad Teaching Hospital during the year 2005. An independent scientific committee revised and approved the study protocol and the information to be provided to the patients. Subjects' written consent was obtained prior to their enrollment in the study. The criterion of inclusion was T2DM. Patients were excluded if they had any previous history of ischemic stroke, hypertension, familial hyperlipidmia, history of angina, myocardial infarction, angioplasty, congestive heart failure, atrial fibrillation coronary bypass, carotid or peripheral vascular surgery, or renal insufficiency. Patients receiving oral hypoglycemic agents, antihypertensive (angiotensin-converting enzyme inhibitors or angiotensin receptor II antagonists) and antiplatelet were not excluded from the study.

A total number of 46 subjects (21 females and 25 males) aged 45 to 77 years were able to comply with the study protocol.

Each patient was clinically examined at the time of initiation of the study. Three blood pressure measurements were taken on the right arm using an appropriately sized cuff with the subject in the seated position. Mean values were taken of the second and third blood pressure readings. Patients with systolic blood pressure ≤ 135 mm Hg and / or diastolic blood pressure ≤ 80 mm Hg were included in the study. Anthropometric measurements of body weight (kg) and height (m) were done. Body mass index (BMI) was calculated using Quetlet's index. Biochemical analysis included fasting plasma glucose, triglycerides, and high density lipoproteins.

Subjects were categorized as having nonhypertensive components of metabolic syndrome when they had at least three of the following criteria:[14,15]

- Body mass index (BMI) ≥ 30 kg/m^2^
- Fasting plasma glucose ≥ 110 mg/dL
- Fasting plasma triglycerides (TG) ≥ 150 mg/dL
- High density lipoprotein (HDL) ≤ 40 mg/dL (men) and ≤ 50 mg/dL (women)

Accordingly, the subjects were assigned to two groups:

- Group I (eight females and nine males): diabetic patients without metabolic syndrome
- Group II (13 females and 16 males): diabetic patients with nonblood pressure component metabolic syndrome

Ultrasonography was performed with B-mode images of a high-resolution ultrasound scanner equipped with a 7 MHz linear array transducer. Anterior, antero-lateral, and postero-lateral projections were used to obtain images of the left and right common and internal carotid arteries.

Arterial diameter and IMT measurements were done three times for each artery at each site. The average of three measurements of each cartotid artery diameter or IMT was taken; the coefficients of variation of these measurements ranged from 1.5 to 2.3%.

### Statistical analysis

The results are presented as absolute number, percent, median, range, and mean ± SD. The data have been analyzed by using unpaired, two-tailed Student's t-test taking *P* ≤ 0.05 as the lowest limit of significance.

## Results

Group II have significantly (*P* < 0.001) high BMI (31.55 ± 3.066, *n* = 29 *vs* 27.49 ± 1.19, *n* = 17), nonsignificant (*P* > 0.05) increases in fasting serum triglycerides (215.1 ± 69, *n* = 29 *vs* 176 ± 105.4, *n* = 17), and significant (*P* < 0.05) decreases in high density lipoprotein (32.8 ± 7.2, *n* = 29 *vs* 39.9 ± 12.1, *n* = 17) than group I patients [Table 1]. No significant differences were observed between group I and II patients in their fasting plasma glucose levels (143.6 ± 13.5, *n* = 17 *vs* 143.8 ± 15.3, *n* = 29 respectively) [Table 1]. However, group II patients have significantly (*P* < 0.02) longer duration of T2DM (13.72 ± 4.8, *n* = 29 *vs* 10.52 ± 3.87, *n* = 17 respectively). The number of smokers was higher in group II than in group I. Common and internal carotid arteries diameters were not significantly different in the two groups [Table 2]. Mean common carotid IMTs (left, right, or both) but not internal carotid artery thicknesses were significantly greater in group II patients compared to group I patients [Table 2]. Group II had a significant number of patients with increased lesion IMT (≥ 1.1 mm) [Table 3].

**Table 1:** Characteristics of the study participants

	Group I (*n* = 17)	Group II (*n* = 29)
Sex		
Male	9	16
Female	8	13
Age (year)		
Male	51 (45–60)	57.5 (49–68)
Female	50 (40–60)	57 (51–77)
BMI (kg/m^2^)		
Male	27.6 (23.2–29.2)	30.4 (27.4–37.7)
Female	28.05 (23.4–29.4)	31.2 (27.3–40)
Total	28.0 (23.2–29.4)	31 (27.3–40)
Fasting plasma TG (mg/dL)		
Male	120 (100–316)	214 (150–300)
Female	133 (100–477)	170 (130–367)
Total	123 (100–477)	200 (100–367)
Fasting plasma HDL (mg/dl)		
Male	36 (29–50)	33 (20–48)
Female	40.5 (26–69)	30 (25–48)
Total	36 (26–69)	30 (20–48)
FPG (mg/dL)		
Male	140 (128–160)	135.5 (122–183)
Female	143.5 (130–177)	146 (129–170)
Total	142 (128–177)	144 (122–183)
Duration of diabetes (year)		
Male	9 (6–18)	14.5 (7–20)
Female	9.5 (6–15)	12 (8–27)
Total	9 (6–18)	13 (7–27)
Smoking (No.)		
Male	3	11
Female	1	4
Total	4	15

The results are expressed as median (range); TG: Triglycerides, HDL: High density lipoprotein, FPG: Fasting plasma glucose

**Table 2:** Mean diameter (mm) and IMT (mm) from common and internal carotid artery measurements

	Group I (*n* = 17)	Group II (*n* = 29)
Diameter (mm)		
Right CCA	6.571 ± 0.608	7.034 ± 1.038
Left CCA	6.723 ± 0.607	6.672 ± 0.790
Both	6.647 ± 0.603	6.853 ± 0.932
Right ICA	4.522 ± 0.623	4.682 ± 0.703
Left ICA	4.664 ± 0.596	4.513 ± 0.636
Both	4.608 ± 0.603	4.598 ± 0.670
IMT (mm)		
Right CCA	0.700 ± 0.127	0.789 ± 0.120*
Left CCA	0.717 ± 0.101	0.858 ± 0.180†
Both	0.708 ± 0.113	0.824 ± 0.155††
Right ICA	0.476 ± 0.160	0.496 ± 0.132
Left ICA	0.470 ± 0.131	0.531 ± 0.187
Both	0.473 ± 0.144	0.513 ± 0.161

* *P* < 0.05,

† *P* < 0.01,

†† *P* < 0.001; CCA: Common carotid artery; ICA: Internal carotid artery; IMT: Intima media thickness

**Table 3:** Number (percent) of cases with increased lesion intima media thickness (≥1.1 mm)

	Group I (n = 17)	Group II (n = 29)
Right CCA	1 (5.9%)	1 (3.4%)
Left CCA	0	5 (17.2%)*
Right ICA	0	0
Left ICA	0	1 (3.4%)

* *P* < 0.05; CCA: Common carotid artery; ICA: Internal carotid artery

## Discussion

Type 2 diabetes mellitus patients with nonblood pressure component metabolic syndrome have significant greater mean common carotid IMTs than those who are free from metabolic syndrome. Significantly greater IMT is observed in the common carotid but not in the internal carotid artery of either side. Many cross studies have shown an association of metabolic syndrome with the thickness of the carotid intima media.[16,17] Our study found that this association of metabolic syndrome in T2DM with the carotid IMT was free of the blood pressure component of metabolic syndrome. This was in contrast to findings by Kovaite *et al.* who reported that blood pressure is the most important factor for significantly high IMT values in metabolic syndrome.[18]

As carotid IMT (a cut-off value of 0.75 mm reported by Holaj *et al*.[21]) is considered to be a marker of generalized atherosclerosis, group II patients may be considered to be at risk for future cardiovascular events[7,19] as well as recurrent ischemic stroke.[20] Carotid IMT is an independent, significant parameter for the prediction of significant coronary artery disease.[21] Accordingly, our patients (group II) are at risk for cardiovascular events because their mean carotid IMT (left or right) is greater than 0.75 mm. Mohan *et al.* reported a higher IMT value (0.95 ± 0.31 mm) in diabetic patients than found in the present study.[22] Possible explanations for this difference may be related to the limited sample size of the current study and the different ethnicity of the study subjects. Moreover, group II was also found to have a significant number of patients with increased lesion IMTs who are also at risk for ischemic stroke. Liou *et al.* found that metabolic syndrome plays a crucial role in the development of recurrent stroke in diabetes patients with an odds ratio of 1.57.[20] The smoking factor may also contribute to the greater IMT in group II. One of the limitations of the current study is the small sample size. We conclude that even in the absence of the blood pressure component, metabolic syndrome in T2DM patients is associated with greater carotid IMT values than in those free from metabolic syndrome. Further prospective studies are recommended to demonstrate the regression of carotid IMT with management of metabolic syndrome in T2DM patients.
